# Tumor suppressor gene alterations in patients with malignant mesothelioma due to environmental asbestos exposure in Turkey

**DOI:** 10.1186/1477-3163-5-23

**Published:** 2006-08-22

**Authors:** Esra Tug, Tuncer Tug, Halit Elyas, Mehmet Coskunsel, Salih Emri

**Affiliations:** 1 Department of Medical Genetics, Abant Izzet Baysal University, Izzet Baysal Medical School, Bolu, Turkey; 2 Department of Chest Diseases; Abant Izzet Baysal University, Izzet Baysal Medical School, Bolu, Turkey; 3 Firat University Medical School, Department of Medical Biology, Elazig, Turkey; 4 Dicle University Medical School, Department of Chest Diseases, Diyarbakir, Turkey; 5 Hacettepe University Medical School, Department of Chest Diseases, Ankara, Turkey

## Abstract

**Background:**

Environmental asbestos exposure can cause the grave lung and pleura malignancies with a high mortality rate, and it is also associated with increased rate of other organ malignancies. Asbestos exposure can develop genotoxic effects and damage in the pleura and lungs.

**Objective:**

In this study, we aimed to determine tumor suppressor gene (TSG) loss in genomic DNA which was isolated from pleural fluid and blood samples of patients with Malignant Pleural Mesothelioma (MPM) due to environmental asbestos exposure.

**Design and patients:**

Prospective study of period from 2001 to 2003 in 17 patients with MPM.

**Methods:**

A total of 12 chromosomal regions were researched by comparing genomic DNA samples isolated from blood and pleural effusion (using PCR, and polyacrilamid gel electrophoresis denaturizing), on 2 different chromosomes which have 9 different polymorphic determinants at 6q and 3 different polymorphic determinants at 9p using molecular genetic methods on 13 patients clinico-pathologically diagnosed MPM.

**Results:**

Loss of Heterozygosity (LOH) was determined at D6S275 in one patient, at D6S301 in another, at D6S474 in 2, at ARG1 in 2, at D6S1038 in 2 and at D6S1008 in 3 patients. In 7 (54%) of the13 patients, we found LOH in at least one site. No LOH was determined at any informative loci in 6 patients. Of the 13 patients, no investigated markers were determined at 9p.

**Conclusion:**

In this study, genomic DNA samples obtained from MPM patients with asbestos exposure revealed that they contained important genotoxic damage. We found no other study on this subject at molecular level in pleural effusion either in Turkey or in the med-line literature. We believe that this study will provide important support for other research into molecular-genetic variations, both on this subject and other malignancies, and may also constitute a base for early diagnosis and gene therapy research in the future.

## Background

Asbestos is a group of naturally-occurring silicate minerals. Silicates are the most abundant minerals (comprising at least 30% of all minerals), which consist of various metals associated with silica and oxygen – the silicate part of the molecule [1]. Asbestos, being an inorganic fibrous mineral, is used in more than 3,000 areas of industry because of its hyper-resistance to high temperatures, friction and chemical agents. An important form of exposure to asbestos is also the inhalation of environmental asbestos dust existing in the geological structures of the some rural settlement areas of the Middle, South-eastern, Eastern and Western Anatolia, and the Black Sea regions of Turkey, since these so-called "white soils" were traditionally used in rural areas to make a whitewash or stucco to surface the walls, floors, and roofs of houses and also as a substitute for baby powder and gripewater [1-4]. Environmental asbestos exposure can cause Malignant Pleural Mesothelioma (MPM), which may be fatal in a very short period, and lung malignancy. It has been reported that the high mortality rate and frequency of MPM, and lung cancer as an endemic disease, are remarkably increased in these areas [1-3,5].

The incidence of MPM has increased world-wide over the last 6 decades and it is assumed that this increase in trend will continue until the year 2020 [6,7].

Mesothelioma develops 3.5 – 73 years (mean 37–40 yrs) after asbestos exposure. Although generally the duration of inhalation and the amount of asbestos dust inhaled are thought to be responsible for the increased incidence of MPM, sometimes an exposure of shorter periods (2–6 months) or lower doses are believed to cause mesothelioma [2]. In a survey achieved in Turkey is reported that the female-to-male ratio was 213:293. The mean age at diagnosis was 56 years (range: 24–88 years), for both men and women. In none of these cases was there a history of occupational exposure to either asbestos or erionite. Six percent of cases (30/506) were reported from the erionite villages [8]. The frequency of malignant mesothelioma is more than 20 times greater among asbestos industry workers and people living in areas with environmental asbestos contact. The frequency of Mesothelioma found in autopsies of asbestos workers is approximately 3%, which is 300 times higher than normal levels [1,2].

It is suggested that multiple, cumulative somatic genetic events are required for tumorigenic conversion of a mesothelial cell [9]. Early studies with conventional banding techniques revealed numerous karyotypic alterations in most MMs [10]. Moreover, structural alterations in all chromosomes, except chromosome Y, were detected [6,11]. Changes consist of recurrent deletions of discrete segments within chromosome arms 1p, 3p, 6q, 9p and 22q [10].

In this study, we aimed to determine LOH in a possible tumor suppressor gene (TSG) region in the pleural fluid samples of patients with MPM contracted, most probably, because of exposure to environmental asbestos in the Elazig Region of Turkey. And thus, the information acquired from the investigation of MPM in molecular-genetic level may also provide illuminated, important somatic genetic mechanisms in both MPM and many other cancer types [6,11].

## Materials and methods

The study group consisted of 17 patients, 8 female (mean age: 61.8 ± 9.8) and 9 male (mean age: 59.4 ± 9.1). The mean environmental asbestos exposure was 27.3 ± 9.1 years. The diagnosis of MPM of all cases was clearly confirmed, both clinically and pathologically. All cases had pleural effusion, had not been treated with cytotoxic chemotherapy, had no malignancy other than MPM, and did not smoke. In our study group, other possible causes of MPM, such as contact with man-made synthetic mineral fiber, asbestiform minerals, beryllium and classical collapse-therapy of tuberculosis or chronic calcified pleural tuberculosis were not determined.

### 2.1. The isolation of genomic DNAs

Genomic DNA isolation was obtained from the patients' pleural effusion by phenol-chloroform methods, and matched with genomic DNA obtained from peripheral blood samples by standard methods. DNA samples obtained by being used to primers such as D6S251, D6S275, D6S301, D6S474, D6S1039, ARG1, D6S1038, D6S441 and D6S1008 primers on the long arm (q) of chromosome 6, and D9S169, D9S126 and D9S171 primers on the short arm (p) of chromosome 9 were amplified by PCR. Localizations of primer and primer series on the chromosomes were obtained from Genome Database [12].

### 2.2. PCR and LOH analysis

PCR was used to amplify genomic DNA in a 25-μl reaction volume containing 2.5 μl 10× Buffer (2 M Tris bas; 1.14 ml glacial ascetic acid; 0.5 M Na_2_EDTA), 1.5 μl MgCl_2 _(25 mM), 1 μl dNTP (2.5 mM), 1 μl forward primer (20 ng/μl) and 1 μl reverse primer (20 ng/μl), 0.1 μl Taq DNA Polymerase (5U/μl), 1 μl genomic DNA and 16.9 μl dH_2_O. PCR was performed in an MJ Research PTC-100 programmable thermal controller. Conditions for the amplification of D6S251, D6S1038, D6S441, D9S126 and D9S169 primers consisted of an initial denaturation for 3 min at 94°C, followed by 32 cycles: 30 sec at 94°C, 30 sec at 53°C and 30 sec at 72°C, and then by a 3 min extension at 72°C. Conditions for the amplification of D6S275, D6S301, D6S474, D6S1039, ARG1, D6S1008 and D9S171 primers consisted of an initial denaturation for 3 min at 94°C followed by 32 cycles: 30 sec at 94°C, 30 sec at 55°C and 30 sec at 72°C, and then by a 3 min extension at 72°C.

PCR products were diluted 1:1 with a 95% formamide gel-loading buffer. Following denaturation at 94°C for 3 min, 7 μl of each sample were size fractionated by electrophoresis through 6% polyacrylamide sequencing gel by 1600 volt current for 3 hours. Silver nitrate dying was applied to gels after electrophoresis and they were photographed with an Automatic Processor Compatible (APC). The allelic patterns obtained from pleural effusions and blood genomic DNAs were compared. One allelic loss in heterozygote individuals was accepted as LOH. LOH was considered to have occurred when there was evidence of 2 alleles in the control DNA and complete loss of an allelic band in the pleural effusion DNA, or when the allelic ratio in pleural effusion DNA differed by a factor greater than 1.6 from the corresponding allelic ratio in control DNA. The intensity of allelic bands was determined by densitometry (NIH Image software, version 1.59).

## Results

Duration of environmental asbestos exposure was not significantly different in the male (mean 27.2 ± 11.2 y) and female (mean 27.4 ± 6.9 y) patients of the study group (p > 0.05). Some demographic features of the patient group are shown in Table 1.

**Table 1:** Malignant pleural, mesothelioma patient characteristics

**Patient No**	**Age**	**Sex**	**Environmental Asbestos Exposure Period (y)**
1	52	Male	25
2	61	Male	20
3	77	Male	40
4	66	Male	20
5	75	Female	40
6	54	Female	20
7	58	Female	18
8	56	Male	25
9	64	Female	30
10	50	Male	50
11	55	Female	30
12	52	Male	20
13	53	Male	15
14	48	Female	25
15	67	Male	30
16	75	Female	30
17	65	Female	26

Comparative molecular studies of 4 patients were not completed: DNA material could not be obtained from patient 15 and patient 16 due to excessive hemorrhagic pattern of pleural fluid samples, and levels of DNA materials obtained from the blood of patient 7 and pleural fluid of patient 17 were not sufficient.

Totally 12 regions selected as 9 regions on chromosomes 6 and 3 regions on chromosomes 9 for LOH analysis. Of the 13 cases of which pleural effusion and blood genomic DNA were compared, 7 (54%) showed allelic loss in 6q. The extent of the partial deletions was used to define 4 discrete minimal regions of nonoverlapping deletion, shortest region overlap 1, 2, 3 and 4 (SRO1, SRO2, SRO3 and SRO4).

The boundaries of the most centromeric region of deletion, SRO1, were defined by marker D6S251 proximally and by D6S249 distally. These represented a region of ~9 cM within 6q14-21. Allelic losses affecting SRO1 were detected in 1 of 13 (8%) MPMs analyzed. SRO2 was deleted in 3 of 13 cases (23%). The distance between D6S301 and D6S474 was ~8 cM, and D6S301 was localized to 6q16.3-21. The 3^rd ^minimally deleted region, SRO3 was defined by an interstitial deletion in 4 of 13 cases (31%). SRO3 was flanked by D6S1039 proximally and by D6S1038 distally. These 2 markers, which are separated by ~10 cM, reside within 6q21-23.2. A 4^th ^discrete region of deletion, SRO4, was defined proximally by the terminal deletion in cases 2, 6 and 10, and distally by deletion in none. SRO4 lies between D6S441 and D6S1008, a region of ~13 cM. Losses affecting this region were observed in 3 cases (23%). On the basis of the position of markers adjacent to D6S441 and D6S1008, SRO4 was probably located within band 6q25.

**Figure 1:**
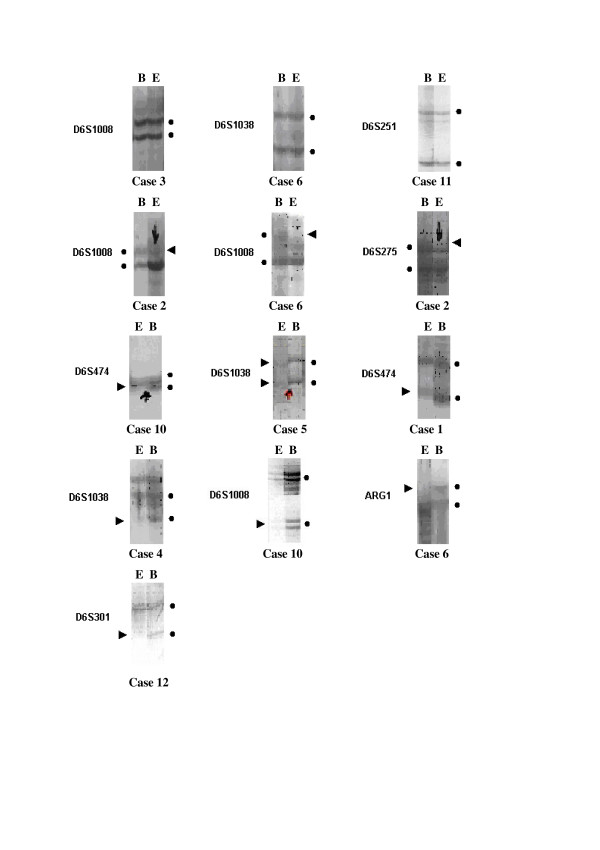
Representative autoradiographs showing allelic band patterns at critical breakpoints that define four minimal regions of deletion of 6q in MPM. It is defined retention of heterozygosity at D6S1008, D6S1038 and D6S251 in case 3, 6 and 11, respectively. Allelic loss at D6S1008 in case 2, 6 and 10; D6S275 in case 2; D6S474 in case 1 and 10; D6S1038 in case 4 and 5; ARG1 in case 6; D6S301 in case 12. Case numbers are shown under the each autoradiograph. Alleles recognized by microsatellite markers are indicated (●). Arrowheads, LOH. B (above panels), normal DNA from peripheral blood; E, pleural effusion DNA.

Of a total of 117 alleles, which were determined by 9 polymorphic determinant for chromosome 6 in 13 patients, 90 (77%) were found to be heterozygote and 27 (23%) homozygote. Individual cases exhibited either a single SRO or more than one SRO (Table 2). Loss of SRO1 alone was not observed. Loss of SRO2 alone was detected in case 12. Losses of both SRO1 and SRO4 were detected in one case (case 2). SRO2 and SRO3 were both lost in case 1. SRO2 and SRO4 were both lost in case 10. Losses of both SRO3 and SRO4 were detected in one case (case 6) (Fig. 1).

**Table 2:** Allelic features and LOH regions found at chromosome 6 in patient group.

**Pat. No**	**Primers**								
	
	**D6S251**	**D6S275**	**D6S301**	**D6S474**	**D6S1039**	**ARG1**	**D6S1038**	**D6S441**	**D6S1008**
1	-	-	-	**+**	-	**+**	±	-	-
2	-	**+**	±	±	-	±	±	-	**+**
3	-	-	-	-	-	±	±	-	-
4	±	±	±	±	-	±	**+**	-	±
5	±	±	-	±	-	-	**+**	-	-
6	±	-	±	-	-	**+**	-	-	**+**
8	-	-	-	-	**±**	-	-	-	-
9	-	-	-	-	-	-	-	-	-
10	±	-	±	**+**	-	±	-	±	**+**
11	-	-	±	-	-	-	-	-	-
12	±	-	**+**	-	-	-	-	-	-
13	-	-	±	-	-	-	-	-	-
14	-	-	-	-	-	-	-	±	-

Pat.: Patient, +: Allelic loss, -: retention of heterozygosity, ± : constitutive homozygosity uninformative. (Due to inability to perform essential PCR analysis patient 7 was not included in the table.)

In 13 patients, of 39 alleles which were determined by 3 markers for 9^th ^chromosome, 18 (46%) were heterozygote and 21 (54%) were homozygote. No LOH case was determined with D9S171, D9S126 and D9S169 markers on 9p (Table 3).

**Table 3:** Allelic characteristics found at chromosome 9p in patient group.

**Patient No**	**Primers**		
	
	**D9S171**	**D9S126**	**D9S169**
1	-	±	-
2	±	±	±
3	±	±	-
4	-	±	-
5	±	±	-
6	±	±	±
8	-	±	-
9	±	±	-
10	-	±	-
11	-	±	-
12	-	±	-
13	±	±	-
14	-	±	-

+: allelic loss, -: retention of heterozygosity, ± : constitutive homozygosity uninformative. (Due to inability to perform the essential PCR analysis patient 7 was not included in the table.)

## Discussion

MPM was observed in 2–10 % of people who had been exposed to high doses of asbestos [6]. The estimated incidence of MPM has been reported to be 43 per million inhabitants in the southeast of Turkey [13]. In asbestos polluted areas of eastern and south-eastern parts of Turkey, mainly tremolite type fibrous mineral exposure was seen [2,14]. The reason for the relative rarity of distant metastases may be related to the rapidity of tumor growth, which generally results in death within 9–10 months after diagnosis. Therapeutic results remain poor and cure of the disease is exceptional [15,16]. Unusually long survival without any treatment is, however, occasionally observed [8].

Epidemiological studies and case reports have revealed that inhaled asbestos dust also increases the frequency of extra pulmonary cancers. The frequency of this kind of pathologies increases with the risk of asbestos exposure. This increase has been determined as 6 times in gastric cancer and 3 times in colon cancer [2,5].

Although there is no common specific chromosomal alteration in MPM patients, inactivation and/or loss of TSG caused by frequent cytogenetic deletions of MPM are thought to be responsible in neoplastic development and progression of mesothelial cells [11]. Complex profiles of characteristic somatic-genetic alterations of MPM show that tumorigenesis of this malignancy is a multi-directional and multi-step period. Karyotype and comparative genomic hybridization analysis of MPM show the presence of deletions in specific regions of 1p, 3p, 6q, 7p, 7q, 9p, 13q, 15q, 17p and 22q chromosome arms [6,12,17].

In MPM, the common sites of allelic losses were in 4 different regions, including 6q14-q21 (SRO1), 6q16.3-q21 (SRO2), 6q21-q23.2 (SRO3) and 6q25 (SRO4) localizations on 6q, and 9p21-p22 region of chromosome 9 [6,11,12]. Loss of function of one or more TSG, determined by LOH at 6q, is interpreted as an important genetic change which contributes development of this malignancy [12].

Bell et al. found higher frequency of losses in 6q (61%) than previously reported ratios (40%) [12]. In our study, LOH was found in at least one site in 7 (54%) of 13 patients. We examined 117 alleles on chromosome 6 and found 90 (77%) of them heterozygote and 27 (23%) homozygote. If we look at the number of examined markers and study material, the ratios we obtained were as successful as other reports on this subject. However, the results of other related studies were derived from DNA samples obtained from tumor derived cell lines. Also it is possible to increase the sensitivity of molecular studies by using DNA extraction from pleural fluid because it is easy, has a low risk profile, and can be repeated rapidly.

Allelic losses for SRO1 were detected in 1 of 13 (8%). SRO2 was also deleted in 3 of 13 cases (23%). SRO3 was defined by an interstitial deletion in 4 of 13 cases (31%), whereas SRO4 was defined in 3 cases (23%).

Recent studies have showed that, in MPM, p16/CDKN2A with 9p21 deletions is an important TSG [11]. Although some important TSG losses in 6q and 9p, which may have an important effect in the development of this malignity, have been reported, the presence of TSG losses at 6q in the early stages of MPM and 9p in advanced stage of MPM are stressed [17].

Although we had determined possible TSG losses at SRO1, SRO2, SRO3 and SRO4 sites of chromosome 6, our inability to determine a loss in p16CDKN2 TSG at region of 9p may be due to the early stages of disease in our patients. Thus, determination of changes that may appear in these 2 chromosomes in the early stages of MPM became a secondary benefit in our study.

Moreover, of 39 alleles of the 13 patients, which were determined with 3 markers for chromosome 9, 18 (46%) were found heterozygote and 21 (54%) were found homozygote. D9S126 was uninformative in all of our cases.

Three or 4 SRO sites of genomic loss from 6q have also been described in other malignancy types, such as breast cancer [18], ovary cancer [19], and Non-Hodgkin's lymphoma [6,11]. In DNA analysis, deletions of interferon locus at 9p21-p22 were also detected in acute lymphoblastic leukemia, glioma, melanoma, lung cancer and bladder cancer [6]. Oncology-consultant research showed no presence of any different tumor.

LOH at p53 TSG accelerates tumor progression [20]. In malignant mesothelioma, it was hypothesized that it may be inactivated by SV40 (simian virus 40) large T antigen (SV40 Tag) [21,22]. SV-40 virus increases the sensitivity of human mesothelial cells against asbestos, causes phenotypic changes and oncogenic transformations in asbestos related lesions, and also, in some rare cases without any relation to asbestosis with malignant transformation, may cause mesothelioma [11]. In the tissue samples derived from MPM patients who had been exposured to asbestos or erionite, no SV40 DNA was found. The reason for this may be because SV40 contaminated vaccines are not used in Turkey [23].

In conclusion, in our study, in patients with MPM, genomic losses at 6q were common, and 4 SRO sites with 6q14-21, 6q16.3-21, 6q21-23.2 and 6q25 deletions were determined. The inability to determine LOH at 9p was explained because of the early stage of MPM of our cases. In our study, contrary to present literature studies on this subject, instead of taking samples from tumor tissue and tumor derived cell lines, we used DNA obtained from pleural fluid which was isolated by thoracentesis. This method is faster, less invasive and more practical than other methods. Molecular genetic clues obtained with more detailed mapping of TSGs in MPM patients may also be collimator for studies aimed at risk, pathogenesis, early diagnosis and treatment of the disease.

## Abbreviations

TSG: Tumor suppressor gene; MPM: Malignant pleural mesothelioma; LOH: Loss of heterozygosity; APC: Automatic processor compatible; SRO1, SRO2, SRO3 and SRO4: Shortest region overlap 1, 2, 3 and 4; SV40 Tag: SV40 Large Tantigen; SV40: Simian virus 40
